# Investigation on Microstructures and High-Temperature Oxidation Resistance of Cr Coatings on Zircaloy-4 by Multi-Arc Ion Plating Technology

**DOI:** 10.3390/ma15196755

**Published:** 2022-09-29

**Authors:** Yingbo Peng, Peinan Du, Yuxi Liu, Haijiang Wang, Shuyu Liu, Wei Zhang

**Affiliations:** 1 State Key Laboratory of Powder Metallurgy, Central South University, Changsha 410083, China; 2 College of Engineering, Nanjing Agricultural University, Nanjing 210031, China; 3 Key Laboratory of Reactor Fuel and Materials, Nuclear Power Institute of China, Chengdu 610213, China

**Keywords:** Cr coating, Zry-4 alloy claddings, EBSD, microstructure, high-temperature oxidation resistance

## Abstract

Introducing the oxidation-resistant coating on Zr alloy is considered to be one of the potential solutions for accident-tolerant fuel (ATF) materials. In this study, pure Cr coatings were prepared on a Zircaloy-4 (Zry-4) alloy surface by multi-arc ion plating under different process parameters. The ability of Cr coating on Zry-4 alloy cladding to improve the oxidation resistance to prevent a loss-of-coolant accident (LOCA) was studied. The microstructure of Cr coating was analyzed using the EBSD technique, and the high-temperature steam oxidation was tested at 800, 1000 and 1200 °C. Compared with the original Zry-4 alloy, the samples with Cr coatings exhibited much better oxidation resistance under different high-temperature steam oxidation conditions. However, the Cr coating exhibited columnar grain, strong preferred orientation and (001) fiber texture. The columnar grain boundaries provided paths for the diffusion of oxygen atoms to the Zry-4 alloy matrix at high temperatures. The results showed that the oxidation film of Cr coating with relatively random grain orientation was compact and uniform and exhibited the best oxidation resistance at high temperatures.

## 1. Introduction

Zirconium alloys have excellent irradiation protection properties, such as a low thermal neutron absorption cross-section, good compatibility with uranium oxide (UO_2_), neutron irradiation resistance and good processability. As a cladding material for fuel rods in the nuclear industry, Zr alloys have been widely used in light water reactors (LWRs) in commercial nuclear power plants [1,2,3]. The zirconium alloy cladding is the sealed shell of the nuclear fuel and worked as the first barrier in protecting the reactor. The main function of zirconium alloy cladding is to contain fission products, isolate fuel rods and coolants, and effectively export the heat energy of nuclear fuel reaction. However, the Zr alloy cladding material can only maintain its protective effect at the normal operation state (only 340 °C position at fuel rod/cladding of the nuclear reactor). Under beyond design basis accident (BDBA) in light water reactors, the coolant water level could drop, thereby exposing the fuel rods, as the nuclear reaction cannot be stopped immediately, a significant rise in core temperature will happen. At this high temperature, Zr alloy claddings oxidize rapidly and lose their protective function on fuel rods. The exposed fuel rods react violently with the coolant and a large amount of hydrogen is generated, resulting in loss-of-coolant accidents (LOCA), e.g., the accident at the Fukushima nuclear power plant in Japan in 2011 [4]. It can be found that the steam oxidation of Zr alloy cladding materials is the main cause of nuclear leakage accidents. The surface modification of Zr alloy cladding is one of the most important approaches to solving the nuclear leakage problem [5]. By introducing the oxidation-resistant coating on the surface of Zr alloy, the contact between Zr alloy and high temperature steam is isolated, preventing the degradation of mechanical properties caused by oxidation and improving the service life of Zr alloy.

At present, some studies on the protective coatings of Zr alloys focused on the metal compound coatings, such as ZrN/ZrO_2_ composite coatings formed by ion implantation [6], TiN coatings by evaporation deposition or laser pulse method [7,8], and some oxides such as Al_2_O_3_, ZrO_2_ and SiO_2_ were also used as a surface modification for Zr alloy cladding [9,10]. Although the above coatings can improve the wear resistance and corrosion resistance of Zr alloy to a certain extent, the metal compound coating has its inevitable shortcomings, especially poor toughness. In the case of the expansion and creep caused by long-term irradiation and high temperatures, the coatings easily crack and peel off, resulting in coating failure and loss of the oxidation-protective effect on Zr alloy.

Among the protective coatings of Zr alloys, Cr and its alloys show excellent mechanical properties, high-temperature oxidation and corrosion resistance, which are ideal materials to replace metal compound coatings. Krejci et al. [11] designed a Cr-based coating on Zr-1%Nb alloy, which can improve the oxidation resistance and mechanical properties significantly and prevent the formation of Cr + Zr eutectic. Using laser additive manufacturing technology, Cr powders were melted on the surface of a Zr-4 pipe to form an 80–200 μm thick Cr coating, and the material exhibited excellent high-temperature oxidation resistance at 1200 °C for 2000 s [12]. Kashkarov et al. [13] prepared a Cr coating on M5_Framatome_ (Zr1Nb) alloy by physical vapor deposition and found that a dense 4.5 μm thick Cr coating provided better oxidation resistance up to 1100 °C. Qing Li et al. [14,15] prepared chromium coating on zircaloy-4 by atmospheric plasma spraying. Compared with zirconium alloy, after oxidation at 1300 °C, the multilayer structure of surface Cr_2_O_3_, residual Cr coating, thin (Zr, Cr) O_2_ layer, Cr Zr layer and zirconium alloy substrate was formed. The oxidation experiment showed that Cr coating can improve the oxidation resistance in a steam environment. Moreover, the grain morphology significantly influenced the oxidation resistance of the Cr coating. E. B. Kashkarov et al. [12] deposited a dense columnar-grain Cr coating on Zr alloy using magnetron sputtering. The thicker coating could better protect against oxidation at 1200 °C. The diffusion behavior of Cr and Zr at the interface significantly affected the oxidation kinetics. Tianguo Wei et al. [16] prepared Zircaloy-4 samples with compact Cr coating using vacuum arc plasma deposition. The Cr coating showed columnar grain structure and grew with the increase in coating thickness, with the size of columnar grain being 1−4 μm. Under the condition of simulating PWR and BWR coolant, the corrosion rate of Cr coating was obviously lower than Zircaloy-4. Therefore, the Cr coatings are considered the most potential accident-tolerant fuel (ATF) coating material in nuclear applications.

Arc deposition technology is ideally suited for coating on cladding materials because it is easy to prepare dense and non-porous coatings with excellent adhesion. Among arc deposition methods, multi-arc ion plating developed by combining evaporation and sputtering technology has been widely used due to the advantages of fast coating speed, good diffraction, good adhesion and high ionization rate [17,18]. In this study, pure Cr coatings were prepared on the surface of a Zr-4 alloy by multi-arc ion plating. By adjusting the parameters of multi-arc ion plating, the coating with different grain morphology and porosity was obtained. The EBSD technique was used to investigate the evolution of microstructure and texture of Cr coatings under different plating processes. By comparing the high-temperature oxidation properties of Cr-coated Zr alloys, the effect of grain morphology and porosity of Cr coatings on the diffusion behavior and oxidation products were studied, in order to improve the ability of Zr alloy cladding material to prevent loss-of-coolant accidents.

## 2. Materials and Methods

### 2.1. Raw Materials and Coatings Preparation

The samples investigated in this study were pure Cr-coated Zr-4 alloy substrate prepared by different multi-arc ion plating processes (Ar atmosphere). As Cr coating deposited on the surface of Zr-4 alloy by multi-arc ion plating, a full factor experiment including temperature, arc current, negative bias, pressure and time was conducted previously. It was found that under the premise of the same coating thickness, arc current and negative bias significantly affected the microstructure and porosity of the coating, while temperature, pressure and time had little effect.

**Figure 1:**
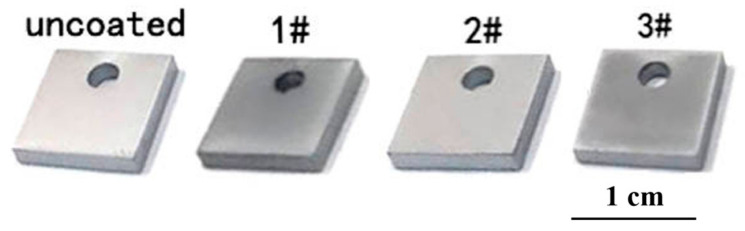
Macro images of uncoated and Cr-coated samples.

When the arc current was less than 120 A, the coating thickness would be significantly reduced. When arc current was higher than 160 A, large droplets from the target formed, which would increase the surface roughness of the coating and significantly deteriorated the properties of the coating. For negative bias, the porosity of the coating decreased with the increase in negative bias. When the negative bias was less than 80 V, the interface between the Cr coating and Zr-4 alloy substrate was mechanically bonded, with a large number of pores. When the negative bias was greater than 160 V, strong interfacial metallurgical bonding was obtained; however, with the further increase in the negative bias, the hardness of the coating increased, and cracks would easily occur. Therefore, in this study, the parameter selection range was 120–150 A arc current and 80–160 V negative bias, as shown in Table 1. The size of Zr-4 alloy substrate was 10 mm × 10 mm × 3 mm with the whole surface coated by Cr, as shown in Figure 1.

### 2.2. Characterization of Coating Samples

EBSD samples were prepared by vibration polishing and argon ion polishing. Vibration polishing was carried out using a Vibromet^®^ 2 vibration polishing instrument (BUEHLER). The ion polishing was operated using an argon ion cross-section polishing instrument (IB–19530CP, JEOL, Tokyo, Japan). The air pressure was controlled at 0.18 MPa, the voltage was 5 kV, and the current was 0.12 μA. The ion polishing started when the vacuum reached 8 × 10^−6^ Pa and the angle of both ion guns was set to 15°. After polishing for 2 h, the voltage was reduced to 3.5 kV and the angle to 12°, and then the polishing process was continued for 1 h more. The microstructure of Cr coatings on the Zr-4 alloy was analyzed using a HKL Channel5 EBSD system equipped with TESCAN MIRA3 field emission scanning electron microscope (SEM) by Oxford Instruments (Abingdon, UK). The EBSD data acquisition step size was 0.1 μm, the working distance was 15 mm, the working voltage was 20 kV, and the sample tilt angle was 70°. The data was processed using Channel5 software (ver. 5.12.57.0).

The microstructure of the coatings was characterized by field emission scanning electron microscopy (FE-SEM, Quanta 650 FEG, Hitachi, Tokyo, Japan). The chemical composition and element distribution were investigated using energy dispersive spectrometry (EDS) matched with SEM. The phase composition was analyzed using an X-ray diffractometer (XRD, D/MAX255) with Cu target Kα radiation, recorded in the 2θ range of 5–80° at a speed of 10°/min.

### 2.3. High-Temperature Oxidation

The high-temperature oxidation tests were carried out with a simultaneous thermal analyzer (AETARAM Instrumentation). The oxidation temperatures of all coating samples were 800 °C, 1000 °C and 1200 °C. The test medium was water vapor with a relative humidity of 90% and the oxidation (holding) time was 2 h. After oxidation tests, the samples were taken out from the instrument and cooled in air. The surface morphology of Cr-coated samples after oxidation was observed by SEM. The weight of coating samples was measured before and after being oxidized at different temperatures to obtain the weight gain due to oxidation. The oxidation resistance of Cr coatings was analyzed using the thermogravimetry (TG) curves.

## 3. Results and Discussions

### 3.1. Microstructures

**Figure 2:**
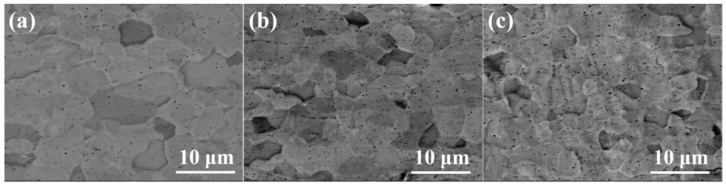
The surface morphology of Cr coating samples: (**a**) 1#, (**b**) 2# and (**c**) 3#.

The SEM images in Figure 2 show the surface of coating samples #1, #2 and #3 (the preparation parameters of each sample were listed in Table 1). The coatings prepared by the multi-arc ion plating process were relatively flat on a macro-scale but exhibited uneven morphology on a micro-scale. The grains appeared to be a near-equiaxed shape, and grain size was almost uniformly distributed in #1 coating, as shown in Figure 2a. In #2 and #3 samples, there were more pits and black particles on the coating surface than #1, as shown in Figure 1b, c. This phenomenon corresponded to parameters of the multi-arc ion plating process [11]. For negative bias voltage, under the action of ion bombardment, original large particles with poor adhesion on the coating surface were easy to desorb from the surface, resulting in some micro pits. For the arc current, with the increase in arc current from 120 A to 150 A, the number of black particles on the surface of the Cr coating increased rapidly, and the surface became rough. This is because the higher arc current will cause the temperature increase in the Cr target, the target will melt around and spray out in the form of droplets, which will deposit on the surface of the substrate to form particles. The higher the temperature, the larger and more particles will be produced.

**Figure 3:**
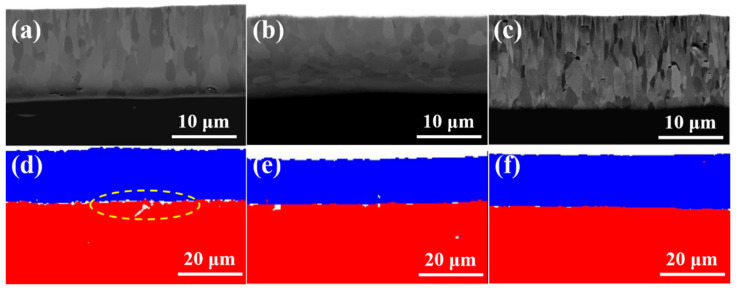
SEM images (**a**–**c**); and phase composition analysis by EBSD (**d**–**f**) of the cross-section of Cr coating samples #1, #2 and #3, respectively. Blue: Cr in the coating part; red: Zr in Zry-4 alloy matrix.

Figure 3a–c show the cross-section microstructures coating samples #1, #2 and #3. It can be seen from the SEM images that the thickness of the coating is 15.1, 14.5 and 15.6 μm, respectively. The coatings can be divided into two regions. From the interface between the Cr coating and Zry-4 alloy matrix, the first was the columnar grain region, and the second was the coarse grain region. In the columnar grain region, the Cr grains presented a columnar or needle-like structure with an obvious preferred orientation. In the coarse grain region, the preferred growth orientation of grains began to be destroyed, and the grains were arranged in a disorderly manner with an obvious increase in grain size. The reason is that the substrate temperature of Zry-4 alloy was not high and the heat transfer was fast in the initial stage of plating. When Cr was deposited on the substrate surface by PVD, the undercooling degree was large and the heat flow perpendicular to the coating thickness direction was provided. Therefore, the grains grew parallel along this direction and formed columnar grain with preferred orientation. With the development of columnar grains to a certain extent, the undercooling degree decreased and the growth rate of grains in different directions tended to be similar, so coarse equiaxed grains were formed. Both 1# and 3# coatings exhibited significantly preferred orientation, and the grains grew along the coating thickness direction, especially for 3# sample with a significant texture, as shown in Figure 2c. The thickness of sample 2# was a little smaller, and the columnar grain growth in 1# and 3# was absent, as presented in Figure 2b. All coatings were closely bonded with the substrate, except sample 1#; the porosity of the 1#, 2# and 3# coating samples were 3.28%, 1.19% and 0.79%, respectively. In addition, the continuous growth of columnar grains in sample 2# and 3# may be disadvantageous to the coating. Because the columnar crystal will cause cracking and spalling under the stress condition, the continuous grain boundaries of columnar crystals are also a defect in the process of oxidation corrosion, which provides channels for oxygen atoms to diffuse into the substrate through the coating.

According to the parameters of multi-arc ion plating, the increase in arc current means an increase in target temperature, and the number, size and temperature of droplets will increase [19]. Therefore, when larger and higher temperature metal droplets are deposited on the Zr-4 alloy substrate, the heat flow direction perpendicular to the substrate surface will be generated locally, and a temperature gradient in the opposite direction of the heat flow will be formed. So, after nucleation on the substrate surface, the grains will grow along the direction of the temperature gradient, forming columnar grains, as shown in Figure 2c, when arc current increased from 120 A to 150 A, the significant columnar grains formed. On the other hand, the negative bias directly influenced the porosity of the coating, and the porosity decreased with the increase in negative bias. When the negative bias was 80 V, the interface between the Cr coating and Zr-4 alloy substrate was almost mechanically bonded, with a porosity of 3.28%. When the negative bias increased to 160 V, strong interfacial metallurgical bonding was obtained, with porosity of 1.19% and 0.79% of 2# and 3# coating sample, respectively, showing good interfacial bonding, as shown in Figure 3d–f. The EBSD phase analysis of the three coating samples, in which Cr in the coating part is shown in blue and Zr in Zry-4 alloy matrix is shown in red.

### 3.2. EBSD Analysis of Cr Coatings

**Figure 4:**
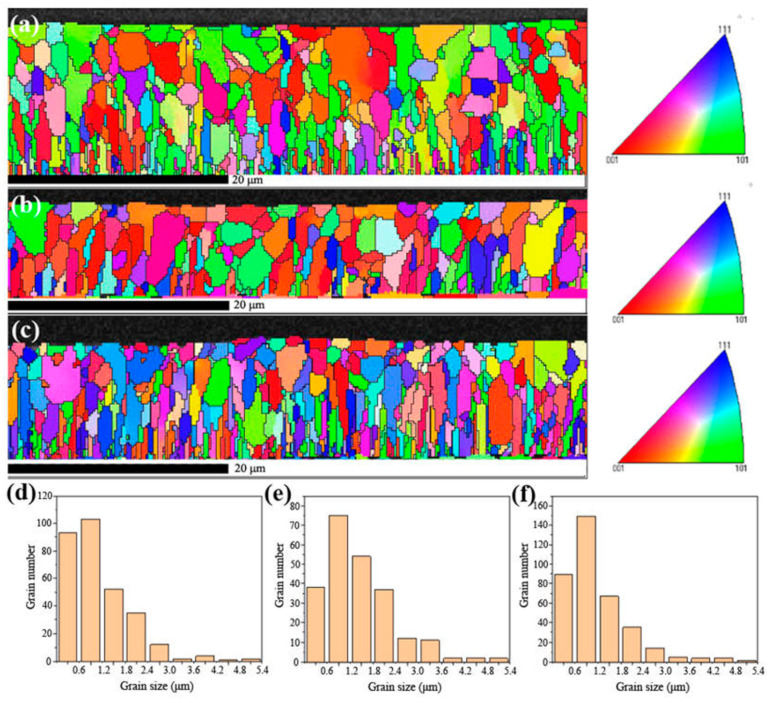
The EBSD reconstruction map and the grain size distribution of the coating samples. (**a**,**d**) 1#, (**b**,**e**) 2# and (**c**,**f**) 3#.

Figure 4 shows the EBSD reconstruction map and the grain size distribution of the coating sample 1#, 2# and 3#. From the EBSD reconstruction map in Figure 4a–c, it can be seen that the grains of 1# and 3# coatings had a strong arrangement along the deposition direction of multi-arc ion plating. The grain orientation of 2# coating was scattered compared with that of 1# and 3#, and there was no obvious preferred orientation.

The grain size of the three coatings was also analyzed in Figure 4e,f. From the bar graphs of grain size and number, it can be found that the grain distribution trend of the three coating samples was consistent, and the grain distribution curves were near-parabolic, reaching the peak value in the range of 0.6–1.2 μm, and more than 90% of the grains were distributed in the range of 0–2.4 μm. The relationship between the grain size of the coating and the distance from the interface also presented a parabola relationship. More than 95% of the grains near the interface between the coating and the substrate were in the range of 0–1.2 μm. The grain size of the coating increased when it was far away from the interface, reached the maximum in the middle region of the coatings, and decreased again after reaching the surface region of the coatings.

The grain size distribution of the three coating samples was similar, and the largest, smallest and the average grain size all exhibited regularity among the coating samples of 2# (1.44 μm) > 3# (1.17 μm) > 1# (1.12 μm). The statistical analysis of grain size is shown in Table 2. Because the grain boundary was a path for elemental diffusion, the smaller the grain boundary was, the more likely it was for hydrogen or oxygen diffusion to form hydride or oxide, causing oxidation or hydrogen embrittlement of the coatings or even the Zry-4 substrate. Therefore, in terms of grain size, the coating sample 2# exhibited more of an advantage than 1# and 3#.

**Figure 5:**
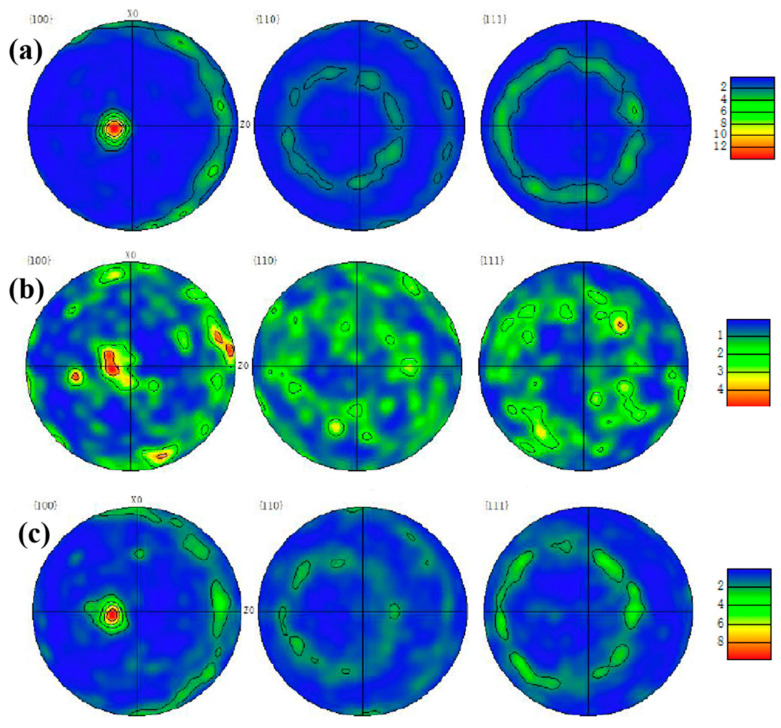
Pole figures of {100}, {110}, {111} planes in the Cr-coating samples. (**a**) 1#, (**b**) 2# and (**c**) 3#.

Moreover, the pole figures of {100}, {110}, {111} crystal planes in the three samples were observed in Figure 5. In the pole figures that consist of iso-density lines, the strength of various orientations can be seen by comparing them with the pole density scale. In the pole figures, the grain orientations of 1# and 3# samples were relatively consistent, as shown in Figure 5a,c. According to their projections on the {100} crystal plane, the grains of both samples exhibited sharp texture on the (001) direction, and the polar density of the texture reached 8–12 grade. In addition, according to the pole figures of {110} {111} crystal planes, 1# and 3# samples are consistent with this and demonstrate no in-plane ordering, as expected. The pole figure of {100} plane in Figure 5b shows that sample 2# exhibited a strong texture on the (101). However, in the {110} and {111} crystal planes, the texture that was parallel to the (011) direction was also obvious, so it was not the real fiber texture and the grain orientation that appeared relatively random compared with 1# and 3# samples.

The grain orientation and morphology of the Cr-coatings are closely related to the oxidation resistance. In the nuclear environment, Zry-4 alloy will absorb oxygen and hydrogen, forming zirconium oxides and hydrides, which shortens the service life of the alloy. The formation of oxides and hydrides is related to the grain morphology of the coating and the habit planes of precipitation. Some studies showed that [12,13] hydrogen atom diffused rapidly into the matrix through the grain boundary, nucleated at the grain boundary of zirconium alloy to form δ-zirconium hydride, and grew rapidly along the grain boundary until it met other grain boundaries.

Compared with the preferred growth of a coating, the columnar grain morphology is responsible for greater diffusivity. A thin film with strong preferred growth but equiaxial grains does not necessarily have diffusion short cuts. A random grain orientation (no preferred growth) with columnar grains will have diffusion short cuts—the oriented grain boundaries. Thus, a large number of columnar grains and the grain boundaries in 1# and 3# coating samples provided the diffusion path of oxygen through Cr coating to Zr-4 substrate. However, this kind of structure was not significant in sample 2#, and the texture of sample 2# was circumferentially distributed. Therefore, from the texture analysis by EBSD, 1# and 3# coatings showed significant fiber texture and distributed along the thickness direction, exhibiting high oxidation tendency. In the reactor environment, the coating was oxidized by high temperature steam, and the oxide formed was related to the grain orientation and morphology of the coating. Oxygen atoms diffused rapidly into the matrix through the grain boundary (columnar grains) of the coating, and the oxide formed grew rapidly along the grain boundary until it met with other grain boundaries. The microstructure of 2# coating was circumferential texture distribution and equiaxial without columnar grains, which reduced the paths of diffusion to Zry-4 substrate, and probably exhibited good oxidation resistance.

### 3.3. High-Temperature Oxidation Resistance

**Figure 6:**
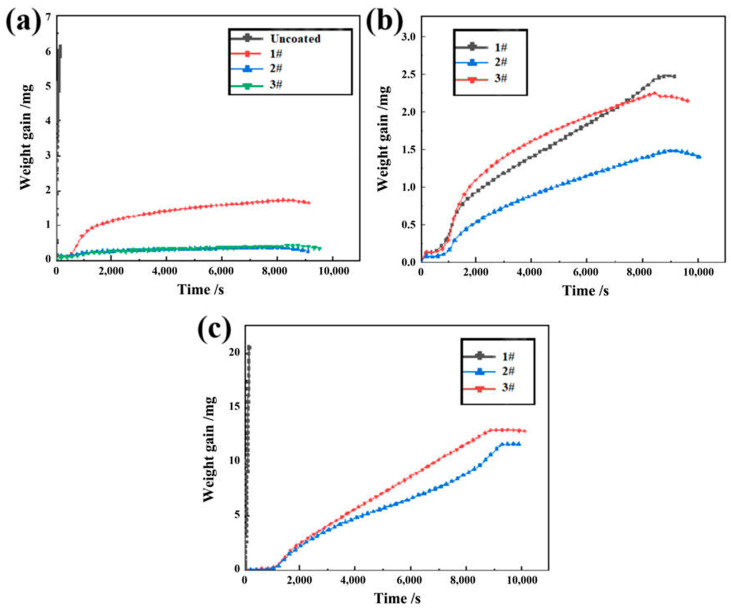
Thermogravimetry curves of three Cr-coating samples oxidized at (**a**) 800 °C, (**b**) 1000 °C and (**c**) 1200 °C.

According to the TG curves of oxidation at 800 °C in Figure 6a, Zry-4 alloy was rapidly oxidized at the initial stage of oxidation only at the minimum testing temperature of 800 °C, and severe oxidation weight gain occurred. However, the weight gain of all three Cr coating samples tended to be stable after slow oxidation, and the oxidation resistance of samples 2# and 3# at 800 °C was better than that of sample 1#. It can be inferred that Cr coatings had better oxidation resistance than Zry-4 alloy substrate at high temperatures and exhibited the protective effect on the substrate. In view of the fact that the Zry-4 alloy substrate had been severely oxidized at 800 °C, it was no longer set as the control group at higher oxidation temperatures.

At 1000 °C, as shown in Figure 6b, the three coating samples still had good oxidation resistance, and sample 2# showed the best protection effect with the minimum weight gain. When the temperature raised to 1200 °C, compared with that at 800 and 1000 °C, the oxidation weight gain of the samples was greatly increased, almost one order of magnitude. Moreover, it can be seen from Figure 6c that the oxidation resistance of sample 1# was rapidly oxidized at the initial stage, and oxidation weight gain increased to peak exponentially in a very short period of time. However, 2# and 3# coatings still exhibited stable oxidation resistance, and the oxidation resistance of 2# coating is better than that of 3#.

Except for the sample with unstable properties (Zry-4 substrate at 800 °C and 1# sample at 1200 °C), taking the oxidation of 2# sample at 1200 °C as an example (blue curve in Figure 6c), the coating was oxidized rapidly at 1200 °C and increased with time. After 9500s, the oxidation weight gain experienced an inflection point, and then the oxidation weight gain remained stable and stopped increasing with time.

**Figure 7:**
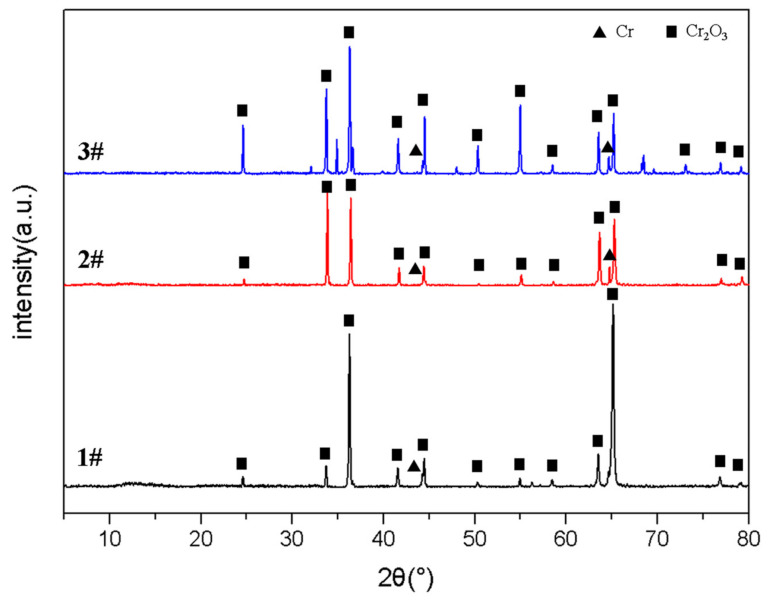
XRD patterns of three coating samples after oxidation at 1200 °C.

From the XRD patterns of coating surfaces in Figure 7, the main product after oxidation is Cr_2_O_3_, together with Cr peaks in all three samples, which indicates that Cr reacts with O_2_ to form Cr_2_O_3_ oxide film. The formation of Cr_2_O_3_ indicates that the coating has good oxidation resistance because Cr_2_O_3_ is insoluble in water, acid/alkali-base corrosive solution [17] and is still inert at high temperatures. On the other hand, there is no Zr or ZrO_2_ peak, which indicates that Cr coating has not failed even at 1200 °C oxidation, and still plays a protective role on the Zr alloy substrate. At the initial stage of oxidation, the free energy of Cr in the coating was negative, the oxidation process was controlled by the formation and growth of Cr_2_O_3_ with a fast growth rate. Thus, the oxidation kinetics showed the accelerated weight gain. With the proceeding of the oxidation process, the element diffusion became the dominant position. The growth rate of Cr_2_O_3_ decreased and tended to be stable. At this stage, it was difficult for oxygen atoms to enter the coating through the original formed oxide film. The rate of oxidation weight gain slowed down. After oxidation for 8000~9500 s, Cr_2_O_3_ had a certain thickness and density, which could inhibit the diffusion of oxygen atoms through the Cr_2_O_3_ film into the matrix, thus preventing the continued growth of the oxide film. Therefore, the oxidation entered a relatively stable stage.

The dense Cr_2_O_3_ oxide film has good protection to Cr coatings. However, for the rapid weight gain of 1# sample during oxidation at 1200 °C (black curve in Figure 6c), it is found from Figure 3a that there were a lot of pores at the interface between the coating and the substrate, which deteriorated the compactness of the formed oxide film, oxygen atoms could still enter the coating or even the Zr alloy substrate through the pores, increasing the oxidation weight gain sharply. In addition, under high-temperature steam, the existence of water vapor accelerated the evaporation of Cr, and the following reactions might occur [18,20]:

2Cr_2_O_3_ + 3O_2_ + 4H_2_O = 4CrO_2_(OH)_2_

The formation of CrO_2_(OH)_2_ led to the continuous loss of Cr, which weakened the formation of the dense oxide layer, which made more oxygen atoms enter the alloy matrix through the oxide film, resulting in the exponential oxidation kinetics curve of 1# sample at 1200 °C.

**Figure 8:**
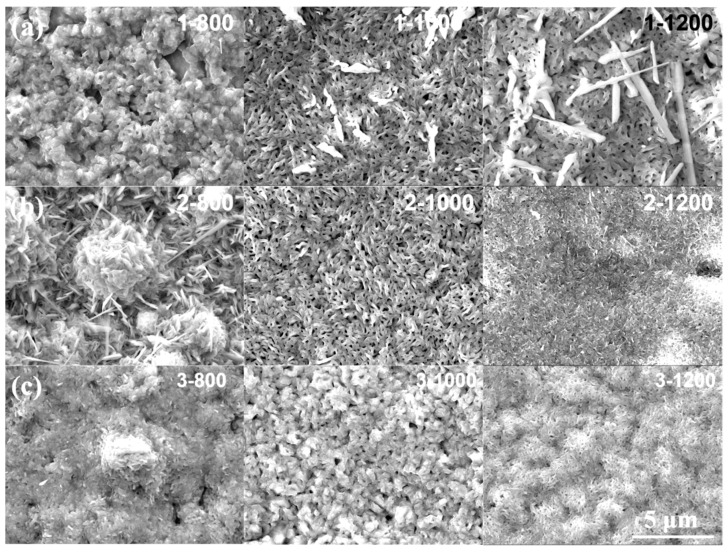
Oxidation surface morphology of coating samples by oxidation at different temperatures. (**a**) 1#, (**b**) 2# and (**c**) 3#.

As shown in Figure 8, during the oxidation of high temperature steam at 800, 1000 and 1200 °C, the three Cr coating samples all exhibited obvious oxidation morphology. The formation of oxide film on the surface of Cr coating is basically a process of nucleation, growth and formation of new oxide on the surface of an existing oxide film.

When oxidized at 800 °C, the oxidation morphology of the three coatings showed a significant correlation with the grain size. In the above analysis of EBSD, the oxidized microstructure of 1# coating sample with the smallest grain size of 1.12 μm was the most uniform and had no agglomeration of oxides (1−800 in Figure 8a). The largest grain size of 1.44 μm of 2# coating showed significant growth and agglomeration of oxides, followed by 3# coating (2−800 and 3−800 in Figure 8a). It is inferred that the nucleation of oxide was at the grain boundary of Cr coating at 800 °C. The structure of the grain boundary was loose due to the irregular arrangement of atoms, so the grain boundary was most vulnerable to thermal and chemical corrosion. In addition, the grain boundary was the channel of rapid diffusion of atoms, and it was easy to cause segregation. That is to say, when pure Cr coating was oxidized in high temperature steam, the fine oxide was formed firstly. With the increase in oxidation time, the fine oxide coarsened, grew, fused and agglomerated; thus, the large granular agglomerated oxides appeared.

After oxidation at 1000 °C, some large-scale flake oxides were precipitated on the surface of 1# sample (1−1000 in Figure 8b), and these large flake oxides were disappeared in 2# sample (2−1000 in Figure 8b). After the oxidation of 3# sample, the oxide agglomeration disappeared and granular oxides appeared which grew and increased obviously, compared with 3−800 in Figure 8c. After oxidation at 1200 °C, the large-scale flake oxides on the surface of 1# sample continued to grow (1−1200 in Figure 8a), and the density of oxide film further decreased, which corresponds to the instability of oxidation resistance of 1# sample. The oxide morphology and density of 2# and 3# samples were similar (2−1200 and 3−1200), showing high density and uniform distribution of oxidation products. It can infer that under the condition of higher temperature, the loose flake oxides appeared on the surface of Cr firstly, and with the proceeding of oxidation, the flake oxides gradually increased, forming dense oxide film.

**Figure 9:**
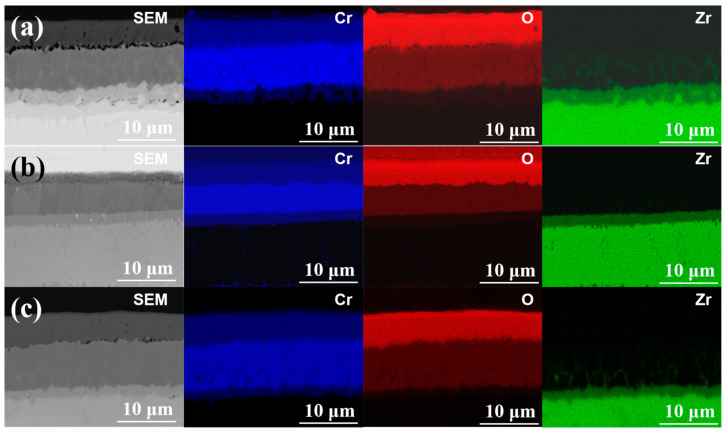
EDS mapping of the longitudinal section of the coatings after oxidized at 1200 °C. (**a**) 1#, (**b**) 2# and (**c**) 3#.

The EDS results in Figure 9 further proved the above analysis. The results show that oxygen of three Cr coating samples did not enter into Zr alloy substrate, but obvious diffusion appeared between sample 1#, 3# and Zr alloy, as shown in Figure 9a,c. The diffusion between Zr alloy substrate and 3# sample was slightly weak, and Zr alloy 2# coating sample was not diffused with Zr alloy, and the interface was smooth and well-bonded, as shown in Figure 9b.

For the effect of parameters, when the arc current increased from 120 A to 150 A, the significant columnar grains formed. The grain boundary of columnar grains will become a direct channel for oxygen atoms diffusion at high temperature, and the oxygen atoms will diffuse to the substrate along the grain boundary perpendicular to the surface of substate. As shown in Figure 6b,c and Figure 9, the steam oxidation resistance at 1000 °C and 1200 °C of 3# coating sample with columnar grain structure, was lower than that of 2# coating sample with equiaxed grain structure. When the negative bias increased from 80 V to 160 V, strong interfacial metallurgical bonding was obtained, with porosity of 1.19% and 0.79% of 2# and 3# coating sample, respectively. At the oxidation temperature of 1200 °C, 1# coating sample with 3.28% of porosity rapidly failed and the oxidation weight gain boosted over 20 mg within 100 s.

**Figure 10:**
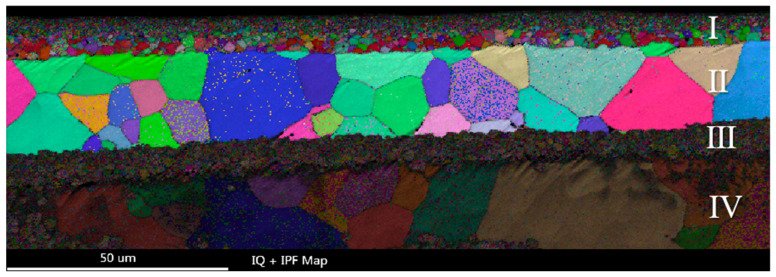
The EBSD reconstructive map of sample 2# after oxidized at 1200 °C. Four layers divided in the longitudinal section of the coating marked I, II, III and IV.

EBSD analysis was carried out on the longitudinal section of 2# sample with smooth, compact and uniform oxide layer at 1200 °C oxidation in Figure 10. The longitudinal section of the coating can be divided into four layers. The layer I was the Cr_2_O_3_ oxide film with refined grains. The Cr_2_O_3_ oxide was equiaxed and the grain size presented gradient distribution. The essence of this microstructure of gradient distributed grains is that the density of grain boundaries (or other interfaces) changes gradiently, so it corresponds to the gradient change of the properties. The gradient change of structure size is different from the simple mixing of different characteristic size structures (such as nano grain, submicron grain and coarse grain). It can effectively avoid the performance mutation caused by structural size mutation. The grains of different size in the gradient structure can be coordinated with each other, and the overall performance and service behavior of the material can be optimized and improved, including corrosion behavior [21,22,23]. Therefore, it can be inferred that the gradient distributed Cr_2_O_3_ oxide grains in the layer I can effectively impede the diffusion of oxygen atoms and improve the oxidation resistance of the coating. The II layer was the initial Cr coating, and the Cr grains were equiaxed without obvious fiber texture. The layer III was the initial Cr-Zr interface region, the microstructure of annealed grains was refined, which was related to the multi-arc ion plating process. The layer IV was the Zr-4 alloy substrate.

Therefore, the three coatings showed good oxidation resistance at 800 °C and 1000 °C. However, with the increase in temperature to 1200 °C, large size oxides were precipitated on the surface of 1# sample, while the oxide film of 2# and 3# coatings became denser, and the oxide film of 2# coating was the densest and uniform with better oxidation resistance.

## 4. Conclusions

In this study, the Cr coating on Zry-4 alloy surface was prepared by different processes of multi-arc ion plating method. The microstructure, grain size and grain orientation of Cr coatings were discussed with EBSD. The oxidation resistance of the coatings at different oxidation temperatures was studied. The main findings are summarized as follows:

(1) The parameters of multi-arc ion plating significantly influenced the microstructure and porosity of the Cr coatings. As arc current increased from 120 A to 150 A, the grain morphology of the coatings changed from equiaxed to columnar. As negative bias increased from 80 V to 160 V, the porosity of the coatings decreased from 3.28% to 0.79%. The average grain size of the coating samples varied from 1.12 μm to 1.44 μm.
(2) The grains of 1# and 3# coating samples exhibited columnar grains along deposition direction and had sharp texture on the (001) crystal plane with fiber features, the polar density of the texture reached 8–12 grade. The sharp texture and columnar grains were caused by the heat flow direction perpendicular to the substrate generated with high arc current. However, there was no obvious preferred orientation structure in 2# sample, and the grains appeared equiaxed.
(3) Compared with the original Zr-4 alloy, the three coating samples exhibited much better oxidation resistance. However, 1# sample lost stability at 1200 °C and the oxidation weight gain increased rapidly due to the micro-pores in the coating, resulting in the deterioration of the densification of the formed oxide film. Large scale flake oxide appeared on the surface of the oxide film, and the compactness of the oxide film was poor.
(4) The oxidation resistance of 2# coating samples was superior, and the oxide film was compact and uniform. Compared with the columnar grains with strong fiber texture in the 1# and 3# sample, which provided paths for oxygen atoms to diffuse into the substrate, the 2# coating sample presented equiaxed grains and reduced or even eliminated the oxygen atom diffusion paths along the grain boundaries of columnar grains. So, 2# coating had the best oxidation resistance.

## Figures and Tables

**Table 1:** The process parameters of multi-arc ion plating.

No.	Temperature (°C)	Arc Current (A)	Pressure (Pa)	Negative Bias (V)	Time (h)
1	300	120	0.8	80	8.5
2	300	120	0.8	160	8.5
3	300	150	0.8	160	8.5

**Table 2:** The grain sizes of the coating samples.

	No.	Mean (μm)	Standard Deviation (SD, μm)	Coefficient of Variation (SD/Mean)
Grain size	1	1.12	0.84	0.75
	2	1.44	0.94	0.66
	3	1.17	0.85	0.73

## Data Availability

The data presented in this study are available on request from the corresponding author.
